# Surgical Resection of Fibrolamellar Hepatocellular Carcinoma After Reassessment of Resectability: A Case Report

**DOI:** 10.1155/cris/6138539

**Published:** 2026-04-25

**Authors:** Ruben R. Lozano-Salazar, Xavier Rios-Covian-Molina, Andrea Rochel-Perez, Osvaldo Huchim-Mendez, Omar Bermudez Ferro, Nina Mendez-Dominguez

**Affiliations:** ^1^ Department of Hepatopancreatobiliary Surgery, Hospital Regional de Alta Especialidad de la Peninsula de Yucatan, IMSS-BIENESTAR, Merida, Yucatan, Mexico; ^2^ Hospital Regional de Alta Especialidad de la Peninsula de Yucatan, Servicios de Salud del Instituto Mexicano del Seguro Social para el Bienestar, Mexico City, Mexico; ^3^ School of Medicine, Anahuac University, Merida, Mexico, anahuac.mx

**Keywords:** carcinoma, fibrolamellar hepatocellular carcinoma, hepatectomy, hepatocellular, liver neoplasms

## Abstract

**Background:**

Fibrolamellar hepatocellular carcinoma (FL‐HCC) is a rare variant of hepatocellular carcinoma (HCC) that typically arises in non‐cirrhotic livers and affects younger individuals compared with conventional HCC. Because patients usually have preserved hepatic function, surgical resection may be feasible even in the presence of large tumors. However, diagnostic uncertainty and concerns regarding extrahepatic disease may limit initial surgical decision‐making.

**Case Presentation:**

We report the case of a 48‐year‐old male patient who presented with epigastric discomfort, weight loss, and a palpable abdominal mass. Contrast‐enhanced imaging demonstrated a large hepatic lesion involving segments II, III, and IV, with additional pulmonary and adrenal findings initially raising concern for metastatic disease. The patient had previously been declined surgical treatment at another institution. Further multidisciplinary evaluation, imaging reassessment, and histological confirmation by percutaneous biopsy supported the diagnosis of FL‐HCC without confirmed metastatic disease. An open anatomical left hepatectomy was performed. Histopathology confirmed FL‐HCC with negative surgical margins and no lymph node metastases. Follow‐up 36 months after surgery demonstrated no evidence of recurrence.

**Conclusion:**

This case highlights the importance of comprehensive staging and multidisciplinary reassessment in patients with suspected FL‐HCC. Even when surgery has initially been declined, careful evaluation may identify candidates for potentially curative hepatic resection. Long‐term surveillance remains essential due to the risk of recurrence following resection. Additionally, this report illustrates that FL‐HCC can occur outside the typical age range, emphasizing the need to consider this diagnosis in compatible clinical and radiologic contexts.

## 1. Background

Hepatocellular carcinoma (HCC) is the most common primary malignant tumor of the liver and represents a major cause of cancer‐related morbidity and mortality worldwide [1–3]. In the majority of cases, HCC develops in the setting of chronic liver disease, particularly cirrhosis related to viral hepatitis, alcohol‐associated liver disease, or metabolic dysfunction–associated steatotic liver disease [1–3].

Fibrolamellar HCC (FL‐HCC) is a rare histological variant of HCC that differs substantially from conventional HCC in its epidemiology, clinical presentation, and biological behavior [4–6]. Unlike classical HCC, which typically arises in cirrhotic livers, FL‐HCC usually develops in adolescents and young adults without underlying chronic liver disease [4–6].

Because hepatic function is generally preserved in these patients, more extensive hepatic resections may be feasible compared with those performed in patients with cirrhosis. The main clinical and epidemiological differences between conventional HCC and FL‐HCC are summarized in Table 1.

**Table 1:** Clinical and epidemiological characteristics of conventional hepatocellular carcinoma and fibrolamellar hepatocellular carcinoma.

Characteristic	HCC	FL‐HCC
Typical age at presentation	40–60 years	20–30 years
Gender	Affects predominantly men	No predilection for sex
Underlying liver disease	Yes, in most cases	No
Tumor markers	High	Normal
Response to systemic therapy	Often responsive	Variable/limited

Abbreviations: FL‐HCC, fibrolamellar hepatocellular carcinoma; HCC, hepatocellular carcinoma.

Although the pathogenesis of FL‐HCC remains incompletely understood, several clinical and molecular characteristics distinguish it from conventional HCC [7–9]. One of the most relevant molecular features is the presence of the recurrent DNAJB1‐PRKACA fusion gene, which has been identified in the majority of cases and is considered a key driver in tumorigenesis [7–9]. Clinically, patients may present with nonspecific symptoms such as abdominal pain, abdominal fullness, weight loss, or the detection of an abdominal mass, and tumors are often large at the time of diagnosis because patients lack underlying liver disease and, therefore, are not typically included in surveillance programs [6, 8].

Despite its rarity, FL‐HCC represents an important clinical entity because its management and prognosis differ from those of conventional HCC. Surgical resection remains the main potentially curative treatment for patients with localized disease and can achieve favorable long‐term survival in selected cases [10]. However, due to the low incidence of the disease, the available evidence remains limited and optimal management strategies are still being defined.

In this report, we describe a case of FL‐HCC in an adult patient who had previously been considered unsuitable for surgical management at another center. This case highlights the importance of careful reassessment of tumor resectability and illustrates relevant diagnostic and surgical considerations in the management of this rare tumor.

## 2. Objective

To describe the clinical presentation, diagnostic evaluation, surgical management, and postoperative outcomes of a patient with FL‐HCC who was initially considered unsuitable for surgical treatment at another institution. In addition, this case aims to highlight key considerations in staging, surgical decision‐making, and follow‐up in the management of this rare hepatic malignancy.

## 3. Case Presentation

A 48‐year‐old male professional musician presented to the hepatopancreatobiliary surgery clinic after being previously evaluated at another institution, where surgical treatment had been declined due to the presence of a large hepatic mass associated with suspected extrahepatic lesions. The patient was therefore initially considered a candidate for palliative management. The patient had a history of tobacco use and heavy alcohol consumption for approximately 15 years, which he had discontinued 10 years prior to presentation. He had no known chronic liver disease, viral hepatitis, metabolic disorders, or previous abdominal surgery. His body mass index was 24.7 kg/m^2^ and his Eastern Cooperative Oncology Group (ECOG) performance status was 0.

The patient reported a progressively enlarging epigastric mass over approximately 1 year, accompanied by burning epigastric discomfort, reduced appetite, and an unintentional weight loss of 10 kg over 2 months. On physical examination, vital signs were within normal limits. Abdominal examination revealed a firm, nontender mass palpable in the epigastrium with limited mobility and well‐defined margins. No jaundice, ascites, or signs of chronic liver disease were observed.

Initial laboratory evaluation demonstrated normal complete blood count and basic metabolic profile. Liver function tests showed mildly elevated alanine aminotransferase (ALT) with normal aspartate aminotransferase (AST) levels. Serum tumor markers were within normal limits, including alpha‐fetoprotein (3.23 ng/mL), carcinoembryonic antigen (2.1 ng/mL), CA 19–9 (5.8 U/mL), and CA 72–4 (0.96 U/mL). Serologic testing for hepatitis B and C viruses was negative.

**Figure 1:**
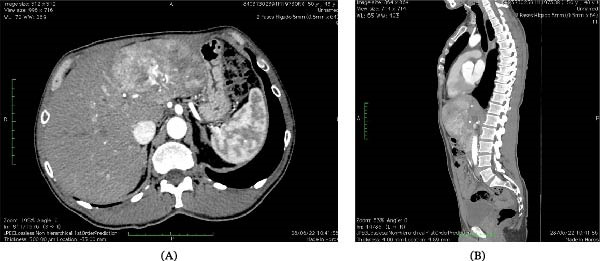
Contrast‐enhanced CT scan of the abdomen. (A) Sagittal section showing the longitudinal extension of the tumor from the T12 to L1 vertebral levels. The anterior displacement of the liver toward the abdominal wall can be observed. (B) Axial section showing the extension of the tumor involving liver segments II and IV. In both sections, the heterogeneous enhancement pattern of the mass can be appreciated.

Contrast‐enhanced computed tomography (CT) of the abdomen demonstrated a heterogeneous hepatic mass measuring 11.3 cm × 11.9 cm × 7.2 cm involving segments II, III, and IV of the liver (Figure 1). The lesion showed features suggestive of FL‐HCC, including a well‐circumscribed mass with heterogeneous enhancement and central scarring.

The CT scan also revealed a right adrenal lesion and small nodules in the right lung base. Because of the concern for possible metastatic disease, additional radiological evaluation and clinical follow‐up were performed. Serial imaging demonstrated stability of these lesions without radiological features consistent with metastatic spread.

A percutaneous Tru‐Cut biopsy of the hepatic lesion was performed, confirming the diagnosis of FL‐HCC.

The case was subsequently discussed at a multidisciplinary tumor board including hepatobiliary surgeons, oncologists, radiologists, and pathologists. After review of the imaging studies and confirmation that the pulmonary and adrenal findings were unlikely to represent metastatic disease, surgical resection was recommended.

Surgery was performed through an open approach using a right subcostal incision with midline extension. After entering the abdominal cavity, a systematic exploration was conducted to rule out peritoneal dissemination or occult metastatic disease. The liver was mobilized by division of the falciform, coronary, and left triangular ligaments. Intraoperative inspection confirmed a large tumor occupying segments II, III, and IV without evidence of vascular invasion or extrahepatic spread.

An anatomical left hepatectomy was performed. Vascular inflow control was achieved using intermittent Pringle maneuvers, consisting of three cycles of 5‐minute inflow occlusion with intervening reperfusion periods, for a total inflow occlusion time of 20 min.

Parenchymal transection was carried out along the plane of the middle hepatic vein using standard clamp‐crush technique with selective vascular ligation and electrocautery hemostasis. The left hepatic artery and left portal vein branches were isolated, ligated, and divided. The left hepatic vein was controlled and divided intrahepatically during parenchymal transection.

Cholecystectomy was performed as part of the procedure. A regional lymph node located in the hepatoduodenal ligament was resected for pathological evaluation. Estimated intraoperative blood loss was approximately 400 mL, and no intraoperative complications occurred.

**Figure 2:**
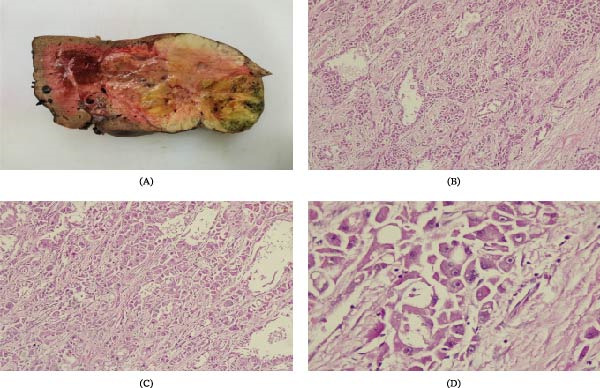
(A) Sagittal section of the removed left liver lobe showing the multinodular mass with a central scar (arrow), close to the anterior surface of the liver. (B–D) Hematoxylin–eosin staining of the tumor specimen. Liver cells are observed with eosinophilic cytoplasm, vesicular nuclei, and prominent nucleoli, surrounded by a fibrous stroma composed of collagen bands arranged in lamellar layers.

Gross pathological examination revealed a 12 cm × 10 cm × 10 cm well‐circumscribed tumor with heterogeneous appearance and a characteristic central fibrous scar (Figure 2A). The surrounding nontumoral liver parenchyma appeared macroscopically normal without nodularity or evidence of cirrhosis. Histopathological evaluation demonstrated large polygonal tumor cells with abundant eosinophilic cytoplasm, prominent nucleoli, and fibrous lamellar bands consistent with FL‐HCC (Figure 2B–D). Microscopic examination of the adjacent nontumoral liver tissue showed preserved hepatic architecture without fibrosis, steatosis, or cirrhotic changes. No lymphovascular or perineural invasion was identified. The resection margins were negative. The regional lymph node removed during surgery showed no evidence of metastatic involvement. The postoperative course was uneventful. The patient was discharged on postoperative day five.

Follow‐up consisted of periodic clinical evaluation, laboratory testing, and imaging studies. Liver function tests and tumor markers remained within normal ranges throughout follow‐up. Contrast‐enhanced CT imaging was performed every 6 months. To date, 36 months after surgery, imaging studies demonstrated no evidence of local recurrence or distant metastasis. Clinically, the patient reported full recovery of baseline functional status, return to professional activity, and restoration of his pre‐illness body weight.

## 4. Discussion

We present the case of a 48‐year‐old man with FL‐HCC, an age that is clearly older than the typical range reported for this tumor. Most clinical series describe FL‐HCC predominantly in adolescents and young adults, usually arising between the second and third decades of life, although cases outside this range have been documented [11–13]. Therefore, while age remains an important epidemiologic clue, it should not exclude the diagnosis when the clinical, radiologic, and histopathologic features are compatible with FL‐HCC.

Another relevant aspect of this case is the history of chronic alcohol consumption. In contrast with conventional HCC, FL‐HCC usually develops in non‐cirrhotic livers and in patients without chronic viral hepatitis or clinically significant underlying liver disease [9, 13, 14]. In our patient, despite prior alcohol exposure, there was no biochemical, radiologic, intraoperative, or histopathologic evidence of cirrhosis or chronic liver disease. This distinction is important because hepatic functional reserve strongly influences surgical planning. In conventional HCC, cirrhosis may limit the extent of resection and may shift treatment toward transplantation or other liver‐sparing approaches. By contrast, in FL‐HCC, preserved liver function often allows major hepatectomy when an adequate future liver remnant is present.

Surgical resection remains the main potentially curative treatment for FL‐HCC and continues to be associated with the best long‐term outcomes when complete tumor removal is feasible [12, 13, 15]. Recent evidence confirms that resection is currently the only established curative option, although recurrence remains common [13]. Reported 5‐year overall survival after resection ranges broadly from approximately 28% to 65%, reflecting differences in stage, nodal status, vascular invasion, completeness of resection, and recurrence patterns [13, 15]. In our patient, preserved liver function and absence of confirmed metastatic disease supported an anatomical left hepatectomy with curative intent.

Regional lymph node status deserves particular attention in FL‐HCC. Lymph node metastases are reported more frequently than in conventional HCC and have been associated with worse prognosis [11, 14, 16]. For this reason, regional nodal assessment may provide relevant staging and prognostic information. In the present case, the regional lymph node examined showed no metastatic involvement, supporting the decision for resection as definitive treatment.

Long‐term surveillance is essential after surgery because recurrence rates remain high, even after apparently curative resection [11, 12]. This justifies structured postoperative follow‐up with cross‐sectional imaging and laboratory assessment. In our case, follow‐up imaging showed no evidence of recurrence at 36 months after surgery, and the patient remains asymptomatic, with full recovery of functional status.

Systemic therapy for FL‐HCC remains poorly defined. Unlike conventional HCC, there is no standard systemic regimen with consistent benefit in FL‐HCC [12–14]. Immune checkpoint inhibitors and other systemic approaches have shown heterogeneous and generally modest activity, although isolated responses have been reported in selected advanced cases [14, 16]. At the molecular level, the recurrent DNAJB1‐PRKACA fusion transcript has emerged as a hallmark of FL‐HCC and may represent a therapeutic target in future strategies [16].

Nonetheless, for patients with localized and resectable disease, surgery remains the cornerstone of management.

This case underscores that FL‐HCC may occur outside the classic age profile and that careful multidisciplinary reassessment can identify candidates for curative resection even when surgery was previously declined elsewhere.

## 5. Limitations

This report has several limitations inherent to case reports. First, the findings are based on a single patient and, therefore, cannot be generalized to the broader population of patients with FL‐HCC. Second, although the patient has remained disease‐free during follow‐up, the observation period is relatively limited, and longer surveillance is necessary to fully assess long‐term oncologic outcomes and potential recurrence. Finally, conclusions regarding optimal management strategies cannot be drawn from an individual case and should be interpreted within the context of existing evidence from larger clinical series and systematic reviews.

## 6. Conclusion

FL‐HCC is a rare primary liver malignancy that typically arises in noncirrhotic livers and may present with large tumors despite preserved hepatic function. Because of its distinct biological and clinical characteristics compared with conventional HCC, careful diagnostic evaluation and multidisciplinary assessment are essential to determine the most appropriate therapeutic strategy.

This case highlights the importance of thorough staging and reassessment of surgical candidacy in patients with FL‐HCC, particularly when surgery has been previously declined. In patients with preserved liver function and resectable disease, hepatic resection remains the main potentially curative treatment and may achieve favorable outcomes.

Long‐term surveillance remains necessary after surgery due to the risk of recurrence, even after apparently curative resection.

## Funding

Institutional funding was received from the Hospital Regional de Alta Especialidad de la Peninsula de Yucatan (Program PP Q008).

## Ethics Statement

Institutional Review Board approval and written informed consent for publication of the case details and accompanying images was obtained from the patient.

## Conflicts of Interest

The authors declare no conflicts of interest.

## Data Availability

The data that support the findings of this study are available upon request from the corresponding author. The data are not publicly available due to privacy or ethical restrictions.
